# Two-year Outcomes of Ventricular-demand Leadless Pacemaker Therapy for Heart Block After Transcatheter Aortic Valve Replacement

**DOI:** 10.19102/icrm.2023.14062

**Published:** 2023-06-15

**Authors:** Daisuke Togashi, Kenichi Sasaki, Kazuaki Okuyama, Masaki Izumo, Ikutaro Nakajima, Taro Suchi, Yui Nakayama, Tomoo Harada, Yoshihiro J. Akashi

**Affiliations:** ^1^Division of Cardiology, Department of Internal Medicine, St. Marianna University School of Medicine, Kawasaki, Japan

**Keywords:** Atrioventricular block, heart failure, leadless pacemaker, mortality, TAVR

## Abstract

Ventricular-demand leadless pacemakers (VVI-LPMs) have often been used as an alternative to atrioventricular (AV) synchronous transvenous pacemakers (DDD-TPMs) in patients with high-grade AV block following transcatheter aortic valve replacement (TAVR). However, the clinical outcomes of this unusual usage are not elucidated. Patients who received permanent pacemakers (PPMs) owing to new-onset high-grade AV block after TAVR from September 2017 to August 2020 at a high-volume center in Japan were included in the analysis, and the clinical courses of VVI-LPM and DDD-TPM implants through 2 years of follow-up were compared retrospectively. Out of 413 consecutive patients who underwent TAVR, 51 (12%) patients received a PPM. After excluding 8 patients with chronic atrial fibrillation (AF), 3 with sick sinus syndrome, and 1 with incomplete data, 17 VVI-LPMs and 22 DDD-TPMs were included in our final cohort. The VVI-LPM group had lower serum albumin levels (3.2 ± 0.5 vs. 3.9 ± 0.4 g/dL, *P* < .01) than the DDD-TPM group. Follow-up revealed no significant differences between the 2 groups in terms of the incidence of late device-related adverse events (0% vs. 5%, log-rank *P* = .38) and new-onset AF (6% vs. 9%, log-rank *P* = .75); however, there were increases in the rates of all-cause death (41% vs. 5%, log-rank *P* < .01) and heart failure rehospitalization (24% vs. 0%, log-rank *P* = .01) in the VVI-LPM group. This small retrospective study reveals favorable post-procedural complication rates but higher all-cause mortality with VVI-LPM compared to DDD-TPM therapy for high-grade AV block after TAVR at 2 years of follow-up.

## Introduction

In accordance with a current guideline,^1^ a ventricular-demand (VVI) leadless pacemaker (PM) (LPM) (VVI-LPM) has been used mainly for atrial fibrillation (AF) with a slow ventricular response (AF-SVR), and the perioperative complications are deemed comparable to those of the VVI transvenous PM (TPM) (VVI-TPM).^2–4^

On the other hand, an LPM could be an attractive therapeutic option for non-AF bradyarrhythmias, such as sick sinus syndrome (SSS) and advanced or complete atrioventricular (AV) block (high-grade AV block), because the device is used in this context to address lead- and pocket-related complications, including vascular occlusion, lead infection, lead fracture, and pocket infection, involved in TPM therapy.^5^ Therefore, it is inevitable that a large number of VVI-LPMs have been implanted in patients with high-grade AV block following transcatheter aortic valve replacement (TAVR), which is a major complication of TAVR, with an incidence of 4%–24%.^6^

Previous articles have reported that dual-chamber TPM (DDD-TPM) implantation for non-AF bradyarrhythmias does not always lead to a better prognosis than VVI-TPM implantation.^7,8^ In contrast, others have stated that DDD-TPM has more advantages in preventing AF, PM syndrome, and exercise intolerance.^9–11^ However, data comparing VVI-LPM and DDD-TPM implantations for non-AF bradyarrhythmias are scarce,^12^ and there are much fewer data available from comparing these therapies for high-grade AV block after TAVR.^13^ Therefore, we sought to examine the clinical courses of patients implanted with a VVI-LPM for high-grade AV block following TAVR and to compare them with those of patients implanted with a DDD-TPM.

## Methods

### Study design and population

**Figure 1:**
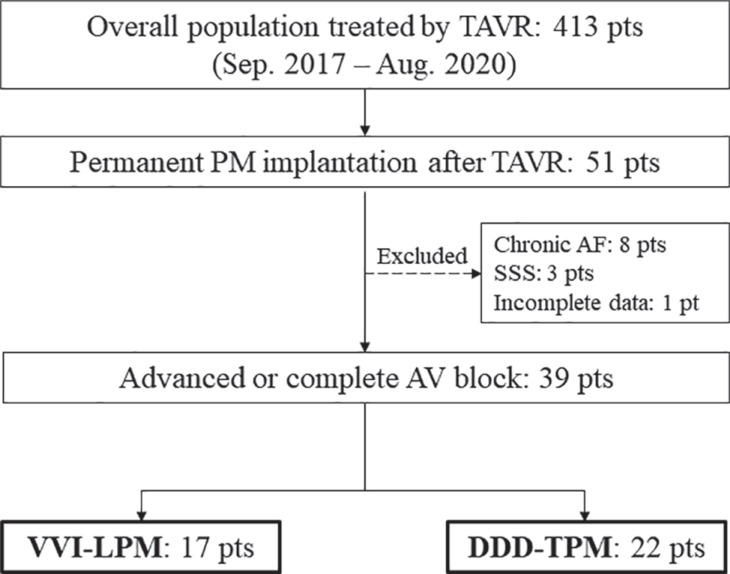
Flowchart of the study population. *Abbreviations:* AF, atrial fibrillation; AV, atrioventricular; DDD-TPM, atrioventricular synchronous transvenous pacemaker; PM, pacemaker; pt, patient; SSS, sick sinus syndrome; TAVR, transcatheter aortic valve replacement; VVI-LPM, ventricular-demand leadless pacemaker.

This was a single-center, retrospective, observational study. We enrolled 413 patients who underwent TAVR between September 2017 and August 2020 at St. Marianna University Hospital (Kawasaki, Japan). Of these, 51 (12%) class I or II patients (according to the Japanese Circulation Society/Japanese Heart Rhythm Society guidelines^1,14^) underwent new permanent pacemaker (PPM) implantation after TAVR. After excluding 8 patients with AF-SVR, 3 with SSS, and 1 with incomplete follow-up data, the final study population consisted of 39 patients with new-onset high-grade AV block, including 17 with VVI-LPMs (45%) and 22 with DDD-TPMs (55%) **(Figure 1)**. The study conformed to the ethical guidelines of the Declaration of Helsinki, and the protocol was approved by the institutional ethics committee (reference no. 5582). Patients were allowed to opt-out of the study.

### Transcatheter aortic valve replacement procedure

The indications for TAVR were discussed and carefully decided by a team that included cardiologists, cardiac surgeons, cardiac anesthesiologists, clinical engineers, and medical radiology technicians. Details on the TAVR procedure are provided elsewhere.^15^ Patients were implanted with a balloon-expandable SAPIEN valve (Edwards Lifesciences, Irvine, CA, USA) or self-expandable Evolut™ valve (Medtronic, Minneapolis, MN, USA). After the procedure, all patients were managed in a general high-care unit for ≥1 day. A temporary PM was placed as a backup for high-grade AV block when necessary. If the AV block persisted or was expected to persist for more than several days after TAVR, attending cardiologists decided whether PPM implantation would be performed during hospitalization.

### Transvenous pacemaker and leadless pacemaker system

Standard techniques were used for DDD-TPM implantation. Based on the attending physicians’ decision, atrial and ventricular leads were positioned in the right atrial (RA) and right ventricular (RV) appendage or septum, respectively. Micra™ (Medtronic) is the only LPM available in Japan. The target implant site was the RV mid-septal region. When this region was found to have inadequate pacing and/or sensing threshold or technical difficulties for deployment, a site in the apical or high RV septum was chosen.

### Electrocardiogram and echocardiography parameters

The 12-lead electrocardiograms (ECGs), used for evaluating the maximum duration of the QRS during RV pacing, and transthoracic echocardiograms (TTEs) were recorded at baseline, 1–6 month(s), 1 year, and 2 years after PPM implantation. During TTE examination, the following data were extracted: left ventricular (LV) ejection fraction (LVEF), LV dimension, the severity of tricuspid valve regurgitation (TR), mitral valve regurgitation (MR), and the estimated RV systolic pressure (RVSP). The LVEF was calculated using the disk summation method. The diameter of the left ventricle was measured at the level of the mitral valve leaflet tips from the parasternal long-axis image. The grading of TR or MR severity was performed according to the American Society of Echocardiography guidelines^16^ and categorized into the following 4 groups: 0 = none or trivial, 1 = mild, 2 = moderate, and 3 = severe. The degree of TR was based on the color flow jet area in the RA observed using the apical 4-chamber view in addition to the continuous-wave Doppler pattern, vena contracta width, and hepatic vein flow. Continuous-wave Doppler of the TR jet was used to estimate the RVSP in consonance with the inferior vena cava size using the modified Bernoulli equation and RA pressure. For the determination of the degree of MR, quantitative data from color Doppler involving the color flow jet area in the left atrium and pulmonary vein flow were used.

In addition, we evaluated the development of the severity of TR and MR, the degree of LVEF reduction, and prosthetic valve function during 1 and 2 year(s) of follow-up. If the severity of regurgitation worsened or improved by ≥2 grades between the value observed at baseline and the value obtained at the 1- or 2-year follow-up (or the last follow-up if the patient died before the annual visit), the severity was defined as “increased” or “decreased,” respectively. A change in severity within 1 grade was defined as “equal.” The substantial reduction of LVEF was defined as a >10% drop compared to baseline. The assessment of prosthetic valve function was based on the Valve Academic Research Consortium (VARC)-3 definitions.^17^

### Nutritional assessment

Prior to the TAVR procedure, the Mini Nutritional Assessment—Short Form (MNA-SF) was used to assess the nutritional problems of elderly patients. The MNA-SF consists of a simple measurement and 6 questions.^18^ The survey collected information on physical measurements (body mass index [BMI], weight loss), global assessment (mobility), dietary questions, and subjective factors (dietary intake, neuropsychological problems, acute illness). According to our recent report that the MNA-SF effectively predicted patient mortality after TAVR,^19^ we focused on this result in the present study.

### Follow-up and endpoints

We evaluated clinical outcomes concerning (1) all-cause mortality, (2) rehospitalization for heart failure (HF), (3) late device-related adverse events (AEs), and (4) new-onset AF during the follow-up period. Basically, clinical events were defined according to the VARC-3 criteria.^17^ Rehospitalization for HF was defined as any new unplanned overnight stay in a hospital because of worsening signs and/or symptoms of HF that required intensification of medical and/or mechanical therapy after PPM implantation. The conclusive diagnosis of HF was made by attending physicians. All late device-related AEs were defined as accidents (eg, device infection, elevated threshold, lead dislodgement, lead failure, or pericardial effusion) that were attributed to the device or implantation procedure and occurred after the patient’s discharge. Elevation of the pacing threshold was defined as an increase by >2-fold from the value when the PPM was implanted. New-onset AF was defined as AF newly documented on a 12-lead ECG during the follow-up period.

Device checks were also performed at 1- and 12-month follow-ups and annually thereafter in an outpatient clinic where the RV pacing burden was acquired. As setting the lower ventricular pacing rate at a higher level led to a higher ventricular pacing percentage (VP%), we adjusted the VP% using the VP% divided by the set lower rate divided by 60 bpm (which was the generally lower rate), thereby calculating the corrected VP% (cVP%). The maximum value of the cVP% was defined as 100%.

### Statistical analysis

Continuous variables are presented as mean ± standard deviation values or median (interquartile range [IQR]) values and were compared using Student’s *t*-test or the Wilcoxon rank-sum test depending on the variable’s distribution. Categorical values are presented as counts and percentages and were compared using Fisher’s exact test. The cumulative incidences of clinical events at follow-up were assessed using the Kaplan–Meier method and log-rank test. Statistical significance was considered achieved at *P* < .05, and analysis was performed using JMP^®^ 14 (SAS Institute, Cary, NC, USA).

## Results

### Baseline characteristics

Comparisons of baseline characteristics between the VVI-LPM and DDD-TPM groups are summarized in **Table 1**. Clinical parameters, such as age, sex, and BMI, were not significantly different between the study groups. Estimated glomerular filtration rate (eGFR) was higher in the VVI-LPM group (71 ± 30 vs. 47 ± 15 mL/min/1.73 m^2^, *P* < .01). It seemed that the higher eGFR in the VVI-LPM group might be associated with (1) the operators’ intention to exclude low-eGFR patients from VVI-LPM implantation because it requires more contrast media or (2) a smaller amount of muscle in VVI-LPM patients, who were malnourished; both the serum albumin level (3.2 ± 0.5 vs. 3.9 ± 0.4 g/dL, *P* < .01) and the MNA-SF score measured preoperatively (5.6 ± 0.8 vs. 8.3 ± 0.8 points, *P* = .03) were significantly lower in the VVI-LPM group.

### Reasons for precluding the use of a transvenous pacemaker

The type of PPM implant (VVI-LPM or DDD-TPM) was determined by the patient’s attending physician or an arrhythmia heart team. Generally, a VVI-LPM was implanted if the following unfavorable conditions for a DDD-TPM were observed: (1) susceptibility to device infection due to the patient’s frailty, steroid use, or other pathology (n = 8); (2) problems with venous access after breast cancer surgery (n = 2); and (3) concern for patients with dementia or delirium who may interfere with the PM pocket (n = 5). In the remaining 2 patients, the indications were based on the physician’s prediction that the treated arrhythmia, like an intermittent AV block, would not require frequent ventricular pacing.

### Electrocardiogram, transthoracic echocardiogram, and procedural parameters

**Table 2** shows comparisons of ECG, TTE, and procedural parameters between the VVI-LPM and DDD-TPM groups. Both groups did not observe a preoperative left bundle branch block pattern. Echocardiographic parameters also did not show significant differences between the 2 groups. Procedure- or device-related AEs during hospitalization occurred in 5 patients (13%). In the DDD-TPM group, 2 cases of pocket hematoma, 1 case of atrial lead dislodgement, and 1 case of elevated pacing threshold were observed, respectively. On the other hand, 1 case of arteriovenous fistula was observed in the VVI-LPM group.

### Changes in echocardiographic and ventricular pacing parameters

Regarding the development of TR and MR and the incidence of reduction of LVEF during 1- and 2-year follow-ups, we found no significant difference between the VVI-LPM and DDD-TPM groups **(Table 3)**. Similarly, no significant differences were observed between these groups concerning post-TAVR prosthetic dysfunctions, such as paravalvular regurgitation and prosthesis–patient mismatch.

In regard to the pacing percentage after TAVR, the cVP% in the VVI-LPM group tended to be lower than that in the DDD-TPM group at the 1-year (52% [IQR, 32%–82%] vs. 75% [IQR, 5%–100%]; *P* = .09) and 2-year (53% [IQR, 62%–100%] vs. 79% [IQR, 8%–99%]; *P* = .07) follow-up visits, but the differences were not statistically significant.

### Clinical outcomes of patients with ventricular-demand leadless pacemaker or atrioventricular synchronous transvenous pacemaker following transcatheter aortic valve replacement

**Figure 2:**
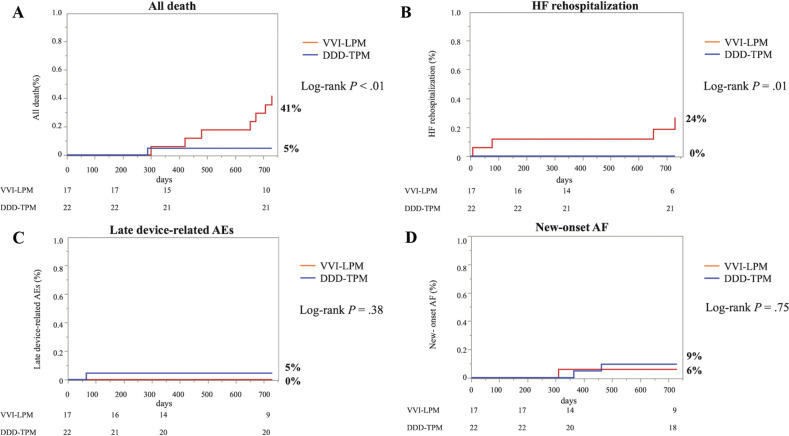
Kaplan–Meier survival curves for **(A)** all deaths, **(B)** HF rehospitalization, **(C)** late device-related AEs, and **(D)** new-onset AF. *Abbreviations:* AE, adverse event; AF, atrial fibrillation; DDD-TPM, atrioventricular synchronous transvenous pacemaker; HF, heart failure; VVI-LPM, ventricular-demand leadless pacemaker.

Clinical outcomes of patients with VVI-LPM or DDD-TPM following TAVR at 1 year of follow-up showed no difference between these groups **(Table 4)**. At 2 years of follow-up, however, there was a higher incidence of all-cause death and HF readmission in the LPM group **(Figure 2)**. During 2 years, all-cause death was observed in 6 cases in the VVI-LPM group and 1 case in the DDD-TPM group (41% vs. 5%, log-rank *P* < .01). Among them, 3 cases in the VVI-LPM group and 1 case in the DDD-TPM group had sudden cardiovascular death. On the other hand, HF rehospitalization occurred in 4 cases in the VVI-LPM group but none in the DDD-TPM group (24% vs. 0%, log-rank *P* = .01). The trigger for HF onset was severe mitral stenosis in 1 case, severe TR in 1 case, LV diastolic dysfunction in 1 case, and AV dyssynchrony in 1 case requiring an upgrade to DDD-TPM therapy. Late device-related AEs were observed in just 1 patient in the DDD-TPM group (5% vs. 0%, log-rank *P* = .38); this patient developed infective endocarditis, which required complete removal of the device system. Finally, new-onset AF was observed in 1 patient in the VVI-LPM group and 2 patients in the DDD-TPM group (6% vs. 9%, log-rank *P* = .75).

## Discussion

Although many previous reports have described the clinical outcomes of VVI-TPM therapy compared to DDD-TPM therapy for high-grade AV block, data on VVI-LPM versus DDD-TPM therapy are scarce and limited in post-TAVR patients. Indeed, the data we previously provided were limited due to the short follow-up duration.^13^ The present study is more informative, offering more precise data regarding clinical outcomes and the changes in AV valve and LV function through a longer follow-up duration. The main findings of this study were as follows. First, (1) the VVI-LPM group had significantly higher rates of all-cause death and HF rehospitalization compared to the DDD-TPM group at 2 years of follow-up; however, there were no significant differences in the incidence of device-related late AEs and new-onset AF. Second, (2) according to the TTE findings, rates of TR and MR onset and the reduction of LVEF through 1 and 2 year(s) of follow-up after PPM implantation were similar between the study groups.

The present study showed that, during the 2-year follow-up period, there were significant differences appeared between the VVI-LPM and DDD-TPM groups in terms of mortality and HF readmission. On the contrary, the majority of previous studies did not demonstrate a better prognosis of DDD-TPM therapy for high-grade AV block than VVI-TPM therapy despite the hemodynamic superiority of DDD pacing.^8,20,21^ The potential reason why patients with VVI pacing had poor clinical outcomes in our study is as follows. As described before, preoperative serum albumin and the MNA-SF score were lower in the VVI-LPM group. The meta-analysis revealed that pre-procedural low albumin levels (cut-off value, 3.5 g or 4 g/dL) were significantly associated with short- and mid-term all-cause mortality in patients after TAVR.^22^ In addition to a lower preoperative serum albumin level of 3.2 ± 0.5 g/dL, the VVI-LPM group had lower MNA-SF scores. Both albumin level and MNA-SF score are useful markers for quantifying nutritional status; thus, it seems reasonable that undernourishment could have an adverse effect on patient prognosis in the VVI-LPM group. Lower albumin levels or MNA-SF scores in the VVI-LPM group might arise from the selection bias by the attending physicians precluding DDD-TPM implantation for apparently frail or malnourished patients. Causes of non-cardiac death, such as infection and cancer, reportedly resulted in a large proportion of deaths after TAVR.^23^ This finding also might confirm our hypothesis that malnutrition was associated with higher all-cause mortality in the VVI-LPM group. Nevertheless, our data observed no significant difference in clinical frailty scores between the study groups. This seems to be due to the inter-rater reliability or small sample size of our study.

The incidence of HF readmission was higher in the VVI-LPM group than in the DDD-TPM group during the 2-year follow-up period. Beurskens et al. suggested that LPM therapy is associated with biventricular and AV valve dysfunction,^24^ while our data did not confirm these findings. As mentioned above, there are conflicting descriptions of worsening cardiac and AV valve function after VVI-LPM implantation.^12,24–26^ To eliminate the discrepancy, we need further studies prospectively investigating TTE parameters with the aid of 3-dimensional echocardiography. Other factors assumed to contribute to the occurrence of decompensated HF, such as wider paced QRS or RV pacing burden, did not differ between VVI-LPM and DDD-TPM therapies in the present study. Therefore, it might be possible that the difference in pacing mode, ie, VVI or DDD, was associated with HF development, although previous studies did not support the hypothesis.^8,20,21^ Then, we focused on the patient’s nutritional condition again. Arquès et al. reported that hypoalbuminemia caused low colloid osmotic pressure and facilitated the onset of pulmonary edema in patients with diastolic HF.^27^ This finding could explain the reason for HF development regardless of LV or AV valve function deterioration.

There were a few late device-related AEs in the present study population. Among DDD-TPM patients, device infection occurred in 1 case at 5 months, and the patient required complete device removal, whereas no late device-related AEs were observed in the VVI-LPM group. It was noteworthy that the VVI-LPM group did not experience any device infection despite their poorer nutrition. It was reported that LPM is more advantageous for patients at high infection risk than TPM because of the resistance to bacteremia.^28,29^ This seems to a reason why the VVI-LPM group did not experience significantly more complications than the DDD-TPM group.

It has been frequently described that VVI pacing for high-grade AV block is associated with a higher incidence of new-onset AF compared to DDD pacing.^10,11^ The reason for increased AF rates with VVI pacing might be because left atrial stretching originating from AV asynchronous RV pacing could increase the pulmonary artery wedge pressure. We did not observe a higher incidence of new AF in the VVI-LPM group; however, this may be responsible for the underestimation of AF due to the inability of LPMs to record arrhythmic events.

Based on these findings, we must discuss whether we should seek to implant DDD-TPMs first in patients with high-grade AV block after TAVR. However, we observed that patients provided with VVI-LPM therapy were likely to be frail and vulnerable; therefore, DDD-TPM implantation in the cohort would result in more device-related AEs and cancel the benefit of physiologic pacing. Considering that the clinical outcomes of both groups were comparable at 1 year of follow-up, we could say that VVI-LPM implantation is reasonable for patients with a limited life expectancy. In any case, it is expected that a recently launched AV synchronous LPM will resolve the dilemma related to device selection, and we should confirm this expectation in the future.

### Study limitation

Our study has several limitations. First, it was a single-center, retrospective, observational study with a relatively small sample size. Thus, the results are likely to be statistically underpowered. Second, there was a selection bias. This study was performed on Japanese post-TAVR patients, so the application of its results to subjects with other clinical backgrounds should be completed with caution. Third, an LPM is incapable of recording arrhythmic events, unlike a TPM, so we cannot ignore the possibility of having underestimated new AF in the VVI-LPM group, especially when AF is asymptomatic. Lastly, the complication rate may have been underestimated because experienced operators in our hospital performed VVI-LPM implantations.

## Conclusion

In this small retrospective study of elderly Japanese patients with high-grade AV block following TAVR, VVI-LPM therapy worsened overall mortality and the HF readmission rate in comparison to DDD-TPM therapy through 2 years of follow-up. Although the results may be affected by different clinical backgrounds, we should continue to be careful when implanting a VVI-LPM in these patients.

## Figures and Tables

**Table 1:** Patient Characteristics

	VVI-LPM n = 17	DDD-TPM n = 22	*P* Value
Age (years)	85 ± 10	85 ± 5	.97
Women, n (%)	13 (76)	17 (77)	.99
BMI (kg/m^2^)	23 ± 3	23 ± 4	.76
Pacing indication			.36
Complete AV block, n (%)	15 (88)	17 (77)	
Advanced AV block, n (%)	2 (12)	5 (23)	
NYHA ≥ III, n (%)	6 (35)	3 (14)	.14
STS score, n (%)	6.3 ± 3.2	5.3 ± 2.7	.30
Clinical Frailty Scale (points)	4.6 ± 1.7	3.9 ± 1.5	.18
MNA-SF (points)	5.6 ± 0.8	8.3 ± 0.8	.03
Hypertension, n (%)	13 (68)	16 (70)	.82
Diabetes mellitus, n (%)	3 (16)	5 (22)	.37
History of AF/AFL, n (%)	2 (12)	1 (5)	.58
History of CAD, n (%)	2 (12)	4 (18)	.58
History of CVD, n (%)	2 (12)	2 (9)	.78
Hemoglobin (g/dL)	10.9 ± 0.3	11.2 ± 0.3	.50
eGFR (mL/min/1.73 m^2^)	71 ± 30	47 ± 15	<.01
Serum albumin (g/dL)	3.2 ± 0.5	3.9 ± 0.4	<.01
NT-proBNP (pg/mL)	3299 (1227–5371)	4647 (970–8324)	.51

*Abbreviations:* AF, atrial fibrillation; AFL, atrial flutter; AV, atrioventricular; BMI, body mass index; CAD, coronary artery disease; CVD, cerebral vascular disease; DDD-TPM, atrioventricular synchronous transvenous pacemaker; eGFR, estimated glomerular filtration rate; MNA-SF, Mini Nutritional Assessment—Short Form; NT-proBNP, N-terminal pro-brain natriuretic peptide; NYHA, New York Heart Association; STS, Society of Thoracic Surgeons; VVI-LPM, ventricular demand leadless pacemaker. Values are expressed as n (%), mean ± standard deviation, or median (interquartile range).

**Table 2:** Electrocardiography/Transthoracic Echocardiography/Procedural Parameters

	VVI-LPM n = 17	DDD-TPM n = 22	*P* Value
**ECG parameters**			
LBBB at baseline, n (%)	0 (0)	0 (0)	1.00
RBBB at baseline, n (%)	8 (47)	14 (64)	.35
Paced QRS width (mm)	156 ± 15	164 ± 15	.10
**TTE parameters at baseline**			
LVEF (%)	66 ± 6	66 ± 7	.98
LVDd (mm)	41 ± 6	44 ± 7	.17
TR ≥ moderate, n (%)	2 (12)	0 (0)	.18
MR ≥ moderate, n (%)	0 (0)	3 (14)	.24
Estimated RVSP (mmHg)	35 ± 13	31 ± 10	.35
**Procedural parameters in TAVR**			
Transfemoral approach, n (%)	17 (100)	22 (100)	1.00
Valve size (mm)	24 ± 3	24 ± 2	.88
Self-expandable valve, n (%)	6 (35)	7 (32)	.99
Peri-procedural complications of PPI	1 (6)	4 (18)	.36

*Abbreviations:* ECG, electrocardiography; LBBB, left bundle branch block; LVDd, left ventricular end-diastolic diameter; LVEF, left ventricular ejection fraction; MR, mitral valve regurgitation; PPI, permanent pacemaker implantation; RBBB, right bundle branch block; RVSP, right ventricular systolic pressure; TR, tricuspid valve regurgitation; TTE, transthoracic echocardiography. Values are expressed as n (%), mean ± standard deviation, or median (interquartile range).

**Table 3:** Changes in Transthoracic Echocardiography Parameters Through 1 and 2 Year(s) of Follow-up^a^

	VVI-LPM n = 17	DDD-TPM n = 22	*P* Value
**1-year or last follow-up**			
MR development, n (%)	1 (6)	1 (5)	.43
TR development, n (%)	2 (12)	3 (14)	.27
LVEF reduction >10%, n (%)	1 (6)	3 (14)	.43
Severe PPM (EOAi < 0.65^b^ or 0.55^c^ cm^2^/m^2^), n (%)	1 (6)	1 (5)	1.00
Moderate to severe^d^ paravalvular regurgitation, n (%)	1 (6)	1 (5)	1.00
RV pacing burden^e^ (%)	52 (32–82)	75 (5–100)	.09
**2-year or last follow-up** ^f^			
MR development, n (%)	0 (0)	0 (0)	1.00
TR development, n (%)	2 (29)	0 (0)	.10
LVEF reduction >10%, n (%)	1 (14)	2 (14)	1.00
Severe PPM (EOAi < 0.65^b^ or 0.55^c^ cm^2^/m^2^), n (%)	1 (14)	0 (0)	.33
Moderate to severe^d^ paravalvular regurgitation, n (%)	0 (0)	0 (0)	1.00
RV pacing burden^e^ (%)	53 (62–100)	79 (8–99)	.07

*Abbreviations:* BMI, body mass index; EOAi, indexed effective orifice area; LVEF, left ventricular ejection fraction; MR, mitral regurgitation; PPM, prosthesis–patient mismatch; RVSP, right ventricular systolic pressure; TR, tricuspid regurgitation; TTE, transthoracic echocardiography. Values are expressed as n (%), mean ± standard deviation, or median (interquartile range). ^a^All data were obtained by comparing values collected at baseline with those collected at each follow-up point. ^b^Used in the setting of BMI < 30 kg/m^2^. ^c^Used in the setting of BMI ≥ 30 kg/m^2^. ^d^Circumferential extent ≥ 10%. ^e^RV pacing burden was replaced by corrected ventricular pacing percentage (cVP%). ^f^Percentages do not equal the number of patients divided by the total number in the study group due to death, censoring, or deficit values.

**Table 4:** One- and 2-year Clinical Outcomes After Pacemaker Implantation^a^

	1-year Event Rates			2-year Event Rates		
	VVI-LPM n = 17	DDD-TPM n = 22	Log-rank *P* Value	VVI-LPM n = 17	DDD-TPM n = 22	Log-rank *P* Value
All death, n (%)	1 (6)	1 (5)	.89	7 (41)	1 (5)	<.01
Cardiovascular death, n (%)	1 (6)	1 (5)	.89	3 (19)	1 (5)	.16
HF rehospitalization, n (%)	2 (12)	0 (0)	.10	4 (24)	0 (0)	.01
Late device-related AEs, n (%)	0 (0)	1 (5)	.38	0 (0)	1 (5)	.38
New-onset AF, n (%)	1 (6)	1 (5)	.76	1 (6)	2 (9)	.75

*Abbreviations:* AEs, adverse events; AF, atrial fibrillation; HF heart failure. ^a^All percentages are Kaplan–Meier estimates at the specific time point and thus do not equal the number of patients divided by the total number in the study group.
